# The optimal transplantation strategy of umbilical cord mesenchymal stem cells in spinal cord injury: a systematic review and network meta-analysis based on animal studies

**DOI:** 10.1186/s13287-022-03103-8

**Published:** 2022-09-02

**Authors:** Yubao Lu, Wei Zhang, Zhenming Tian, Qian Liang, Chenrui Liu, Yingjie Wu, Liangming Zhang, Limin Rong

**Affiliations:** 1 Department of Spine Surgery, The Third Affiliated Hospital, Sun Yat-Sen University, No.600 Tianhe Road, Guangzhou, 510630 Guangdong China; 2 National Medical Products Administration (NMPA) Key Laboratory for Quality Research and Evaluation of Cell Products, Guangzhou, 510630 China; 3 Guangdong Provincial Center for Quality Control of Minimally Invasive Spine Surgery, Guangzhou, 510630 China; 4 Guangdong Provincial Center for Engineering and Technology Research of Minimally Invasive Spine Surgery, Guangzhou, 510630 China

**Keywords:** Spinal cord injury, Umbilical cord mesenchymal stem cells, Therapeutic strategies, Animal study, Systematic review, Network meta-analysis

## Abstract

**Objective:**

Umbilical cord mesenchymal stem cells (UCMSCs) have great potential in the treatment of spinal cord injury. However, the specific therapeutic effect and optimal transplantation strategy are still unclear. Therefore, exploring the optimal treatment strategy of UCMSCs in animal studies by systematic review can provide reference for the development of animal studies and clinical research in the future.

**Methods:**

Databases of PubMed, Ovid-Embase, Web of Science, CNKI, WanFang, VIP, and CBM were searched for the literature in February 11, 2022. Two independent reviewers performed the literature search, identification, screening, quality assessment, and data extraction.

**Results and Discussion:**

A total of 40 animal studies were included for combined analysis. In different subgroups, the results of traditional meta-analysis and network meta-analysis were consistent, that is, the therapeutic effect of high-dose (≥ 1 × 10^6^) transplantation of UCMSCs was significantly better than that of low dose (< 1 × 10^6^), the therapeutic effect of local transplantation of UCMSCs was significantly better than that of intravenous transplantation, and the therapeutic effect of subacute transplantation of UCMSCs was significantly better than that of acute and chronic transplantation. However, in view of the inherent risk of bias and limited internal and external validity of the current animal studies, more high-quality, direct comparison studies are needed to further explore the optimal transplantation strategy for UCMSCs in the future.

**Supplementary Information:**

The online version contains supplementary material available at 10.1186/s13287-022-03103-8.

## Introduction

The annual incidence of spinal cord injury (SCI) varies widely worldwide, ranging from 13.0 to 163.4 cases per million people, and can lead to permanent deterioration of motor, sensory, and autonomic function of the central nervous system, which in turn leads to paralysis, paresthesias, cramps, pain, and cardiovascular, bowel, bladder, or sexual dysfunction [1–3]. The main causes of SCI are traffic accidents (38%), falls (31%), and sports injuries (10–17%) [4]. After SCI, a series of continuous pathophysiological injuries occur, such as direct injury leading to ischemic necrosis of the spinal cord [5], intracellular calcium accumulation [6], and biochemical changes such as lipid peroxidative damage and accumulation of excitatory amino acids [7], eventually leading to demyelination, Wallerian degeneration, oligodendrocyte apoptosis, and glial scarring [8, 9]. Therefore, traditional treatments, such as drug therapy (methylprednisolone, erythropoietin, riluzole, minocycline), hypothermia therapy, and surgery, can only alleviate the further development of SCI and improve the quality of life of patients, with limited effect on the recovery of patients function [10, 11].

In recent years, the progress of stem cell transplantation technology and the in-depth research on the pathophysiology of SCI have brought new hope to patients with SCI. Stem cells can promote functional recovery of injured spinal cord by replacing damaged neurons, promoting remyelination of axons, promoting angiogenesis, bridging cysts or cavities, and reducing inflammatory factors [12]. Umbilical cord mesenchymal stem cells (UCMSCs) are easily obtained from cord blood, perivascular and subendothelial umbilical veins without ethical issues, and in addition, UCMSCs have been shown to have superior differentiation, migration, and protection properties than other types of stem cells [13]. Therefore, UCMSCs have attracted much attention in the treatment of SCI. Preclinical animal studies have demonstrated the great therapeutic potential of UCMSCs [14, 15]. However, the clinical translation of UCMSCs is not smooth. For example, Dai et al. directly transplanted autologous UCMSCs to the site of SCI and found that only 8 of the 18 patients had mild recovery of sensory and motor functions, and stem cell transplantation may cause adverse reactions such as fever, headache, and neuropathic pain [16]. The phase 3 clinical trial of Oh et al. also showed that of the 16 patients who received stem cell transplantation, only 2 patients showed a slight improvement in neurological function [17]. The main reason that hinders the clinical translation of stem cell therapy is that the optimal transplantation route, dose, and timing of stem cell transplantation strategies are unclear [18]. However, there are few animal studies to explore the optimal transplantation strategy of stem cells. At the same time, limited to safety and ethical issues, it is even more unrealistic to explore the optimal repair strategy of stem cells in clinic.

As the first systematic review in the current field, this study intends to comprehensively collect published animal studies to explore the optimal transplantation strategy for UCMSCs, in order to improve their therapeutic effect and provide references for future animal and clinical research.

## Materials and methods

### Inclusion and exclusion criteria

#### Patients and Diseases (P)

A rat model with SCI.

#### Interventions (I)

Umbilical cord mesenchymal stem cells (UCMSCs), with a single transplantation.

#### Control (C)

①Positive controls: Comparison of different transplantation routes, doses, and timings of UCMSCs. ②Negative controls: Blank, DMEM, PBS, Saline, Vehicle.

#### Outcomes (O)

Basso–Beattie–Bresnahan (BBB) locomotor rating scale [19]. The BBB scores represent a detailed and ordinal categorization of hindlimb locomotor recovery after spinal cord injury.

#### Type of study (S)

Control studies were included.

#### Exclusion criteria

(1) Unreported studies on the transplantation route, timing, and dose of UCMSCs. (2) Studies that did not report BBB scores. (3) Reviews, conference abstracts, case reports, clinical trials, etc.

#### Data selection

Astrocytes can form glial scar in the third week after SCI, thereby hindering nerve regeneration [20]. In addition, the recovery of motor function in rats after SCI appears a plateau at about 5 weeks [21, 22]. Therefore, we selected the third- and fifth-week data for analysis.

### Data sources and searches

Candidate studies were identified through searches of PubMed, Ovid-Embase, Web of Science, China National Knowledge Infrastructure (CNKI), Chinese Scientific Journal Database (CSJD-VIP), WanFang Database, and China Biomedical Literature Database (CBM) databases from their inception until February 11, 2022. The following terms were combined to design the search strategy: (“Spinal cord injury” OR “Spinal injury” OR “Spinal Cord Trauma” OR “Spinal Cord Transection” OR “Spinal Cord Laceration” OR “Post-Traumatic Myelopathy” OR “Spinal Cord Contusion”)) AND (“umbilical cord mesenchymal stem cells” OR “umbilical cord stem cells” OR (umbilical cord AND (“stem cells” OR “stem cell”)) OR UCMSCs). Further details of the search strategy are shown in Additional file 1: Table S1.

### Literature screening and data extraction

Two trained researchers selected the papers and stringently extracted the data based on the inclusion/exclusion criteria, and the selections were cross-checked. In the case of disagreement, a third researcher settled the conflict with a common consensus. Data were extracted according to the pre-established full-text data extraction checklist, including (1) basic characteristics of studies such as authors, publication years, type of study, baseline characteristics of rats (gender, age, weight), sample size, modeling method, source of UCMSCs, transplantation route, dose, timings, and controls. (2) Key elements of bias risk assessment. (3) Outcome measures: BBB score.

### The risk of bias among included studies

Based on SYRCLE's risk of bias tool for animal studies [23], two trained researchers independently evaluated and cross-checked the inherent risk of bias in the included studies, covering selection bias, implementation bias, measurement bias, follow-up bias, report bias, and other bias from a list of 10 questions or tools. A difference in opinions was negotiated or decided by a third party. The answer to the assessment questions (tools) should be either “yes” that indicated low risk of bias, or “no” that indicated high risk of bias. For unclear items, an answer with “unclear” was assigned.

### Statistical analysis

WinBUGS 1.4.3. is used for data analysis. For measurement data, standardized mean difference (SMD) is used as the effect statistic index and its 95% confidence interval (95% CI) was calculated. Initial values are set using four Markov chains. The number of iterations for the initial update of the model is set to 10,000, and the number of iterations for continuous update is set to 100,000. The first 10,000 annealing times are used to eliminate the influence of the initial value, and sampling starts after 10,001 times. When there is a closed loop, the consistency between direct comparison and indirect comparison is judged by the node split value, and the inconsistency is considered obvious when *P* < 0.05. The likelihood of each intervention is present being the best intervention through a ranked probability plot. Graphics is drawn using Stata16.0.

### Subgroup analysis

In order to avoid the influence of confounding factors (such as severity of SCI, different transplantation dose, route, and timing) on the results of meta-analysis, and to reduce the heterogeneity among the included studies, we analyzed the included studies by subgroups. (1) Transplantation dose: high dose (≥ 1 × 10^6^) and low dose (< 1 × 10^6^). (2) Transplantation route: local transplantation (direct transplantation of UCMSCs into the spinal cord parenchyma or intrathecal transplantation into the subarachnoid space) and intravenous transplantation. (3) Transplantation timing: acute phase (≤ 3 days), subacute phase (≤ 14 days), and chronic phase (> 14 days) [6, 24]. (4) Severity of SCI: minor (contusions and compression injuries), moderate (hemi-transected injury), and severe injuries (complete transection injury).

## Results

### Systematic search outcomes

**Fig. 1:**
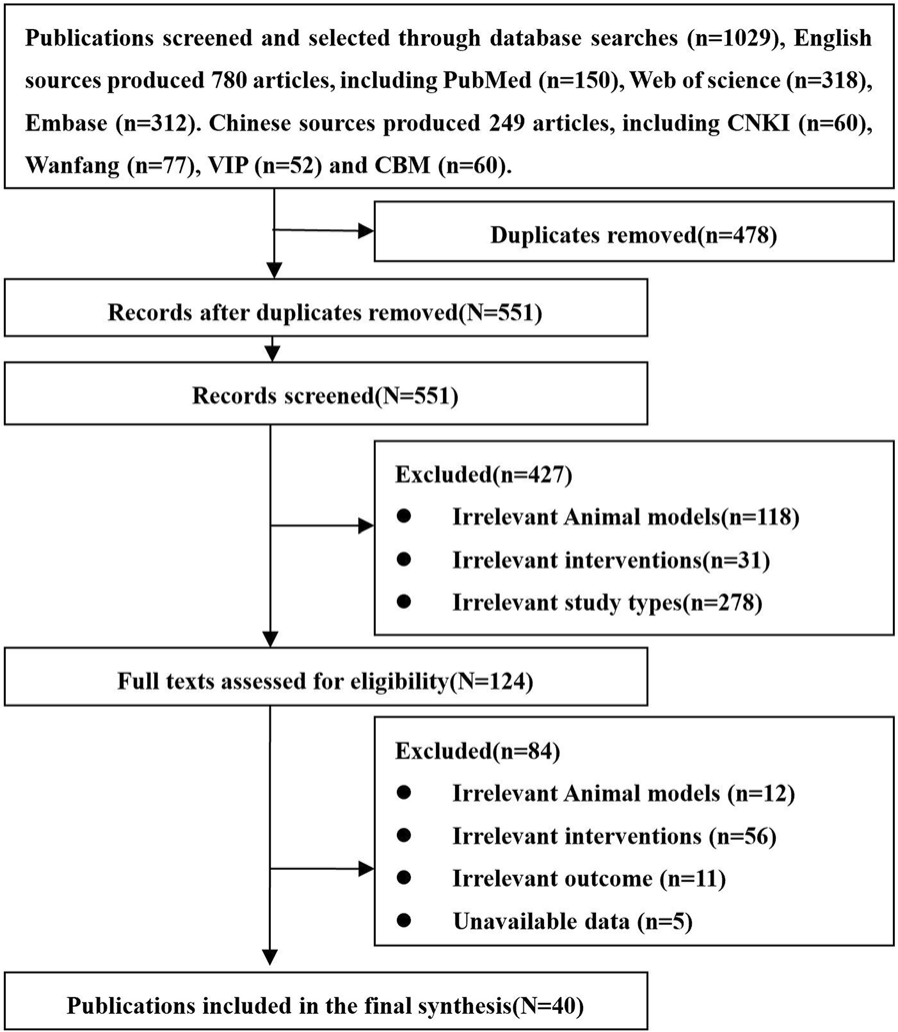
PRISMA flowchart

A total of 1029 articles were retrieved, including 780 English articles and 249 Chinese articles. After excluding repetitive articles, irrelevant animal models (rabbits, monkeys, etc.), irrelevant interventions (UCMSCs-derived exosomes, cytokines, cord blood stem cells, etc.), and irrelevant study types (reviews, statements, reports, opinion papers, comments, editorial, and conference abstracts), 40 animal studies were finally included, including 19 English articles and 21 Chinese articles. The PRISMA flowchart describing the inclusion process is presented in Fig. 1.

### Basic information for inclusion in the study

All 40 included animal studies were randomized controlled trials. The species of rats included SD rats (26 studies) and Wistar rats (14 studies). The gender of rats included male (9 studies), female (28 studies), half male and half female (1 study), of which 2 studies did not report the gender of rats. Rats weighed between 140 and 305 g and aged between 6 and 14 weeks, sample size being between 12 and 90. Model types include contusion (30 studies), compression (6 studies), transection (1 study), and hemi-sectioned (3 studies). Stem cells were derived from umbilical cord tissue of human (38 studies), SD rats (1 study) and Wistar rat (1 study). The transplantation dose was between 6 × 10^3^ and 1.5 × 10^7^, whereas that in the low-dose group was between 6 × 10^3^ and 6 × 10^5^ and in the high-dose group was between 1 × 10^6^ and 1.5 × 10^7^. The transplantation route included local transplantation (29 studies) and intravenous transplantation (11 studies); transplantation timing was between 0 and 21 days after modeling. Controls included Blank (10 studies), DMEM (19 studies), DMSO (1 study), PBS (4 studies), and saline (6 studies). The basic information of the included studies is shown in Additional file 1: Table S2.

### Risk of bias assessment results

**Fig. 2:**
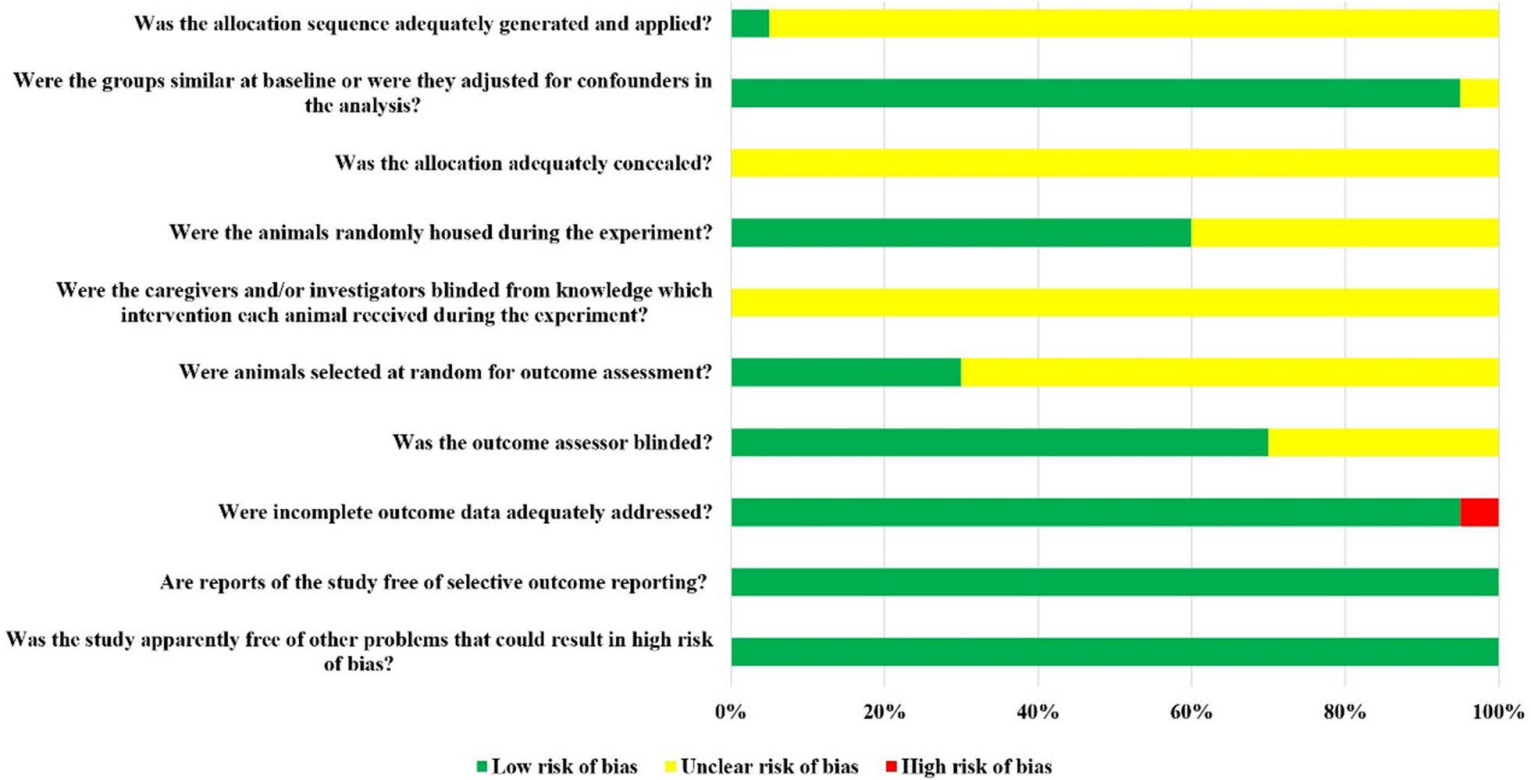
Risk of bias assessment results

Although the included 40 studies were all randomized controlled trials, only 2 studies reported randomization of animals using a random number table, but they did not report whether concealed grouping was performed. Thirty-eight studies clearly reported that the baseline characteristics of laboratory animals, such as age, sex, and weight, were balanced. Twenty-four studies reported randomized placement of animals during the experiment. Due to the limited information provided, all included studies were unable to determine whether animal breeders and/or investigators were blinded. Only 12 studies reported random selection of animals at the time of outcome measurement. Blinding results in evaluators in 28 studies. All the animals in the 38 studies were included in the final analysis. All studies clearly reported all expected results, although research protocols were not available. The bias risk assessment results of included studies are detailed in Fig. 2.

### Meta-analysis results

Among the 40 studies included, there were 36 studies in minor-injury group, 3 in moderate-injury group, and 1 in severe-injury group. A network meta-analysis was not possible due to the limited number of studies in the moderate- and severe-injury groups, which did not constitute a network, so we only performed a traditional meta-analysis for them. Both traditional and network meta-analyses were performed for the minor-injury group.

The results of meta-analysis in moderate- and severe-injury groups showed that regardless of injury model or stem cell transplantation strategy, UCMSCs could significantly improve motor function in rats with SCI compared with placebo group (Table 1).

**Table 1:** Traditional meta-analysis results of moderate and severe spinal cord injury

Group	Number of studies	The third week (SMD)	The fifth week (SMD)
Moderate injury + Acute phase + Intravenous transplantation + High dose	2	4.74 (3.04, 6.44)	7.07 (4.70, 9.44)
Moderate injury + Acute phase + Local transplantation + Low dose	1	2.73 (1.56, 3.90)	3.09 (1.84, 4.34)
Severe injury + Subacute phase + Local transplantation + Low dose	1	4.76 (3.01, 6.51)	10.20 (6.84, 13.56)

#### The results of traditional meta-analysis of optimal transplantation dose (minor SCI)

When exploring the optimal dose of stem cell transplantation, we divided the included studies into four subgroups: local transplantation + acute phase transplantation group; local transplantation + subacute phase transplantation group; intravenous transplantation + acute phase transplantation group; and intravenous transplantation + subacute phase transplantation group. Since only high-dose stem cell transplantation was performed in the intravenous transplantation + subacute phase transplantation group, the transplantation effect was not compared with that of low-dose stem cells. The results of traditional meta-analysis at different time points in the other three groups all showed that stem cells can significantly improve the motor function of rats with SCI, and the therapeutic effect of high-dose stem cell transplantation is better than that of low-dose stem cell transplantation (Table 2).

**Table 2:** Traditional meta-analysis results of optimal transplantation dose

Group	Dose	The third week (SMD)	The fifth week (SMD)
Local transplantation + acute phase transplantation	High dose	2.93 (0.90, 4.96)	6.38 (2.96, 9.81)
	Low dose	2.22 (1.23, 3.22)	2.83 (1.56, 4.11)
Local transplantation + subacute phase transplantation	High dose	2.92 (1.80, 4.05)	5.42 (3.36, 7.47)
	Low dose	1.49 (0.48, 2.51)	2.56 (0.82, 4.31)
Intravenous transplantation + acute phase transplantation	High dose	2.57 (0.90, 6.03)	4.63 (2.83, 6.43)
	Low dose	1.60 (0.91, 2.29)	1.80 (0.30, 3.30)

#### The results of network meta-analysis of optimal transplantation dose (minor SCI)

**Fig. 3:**
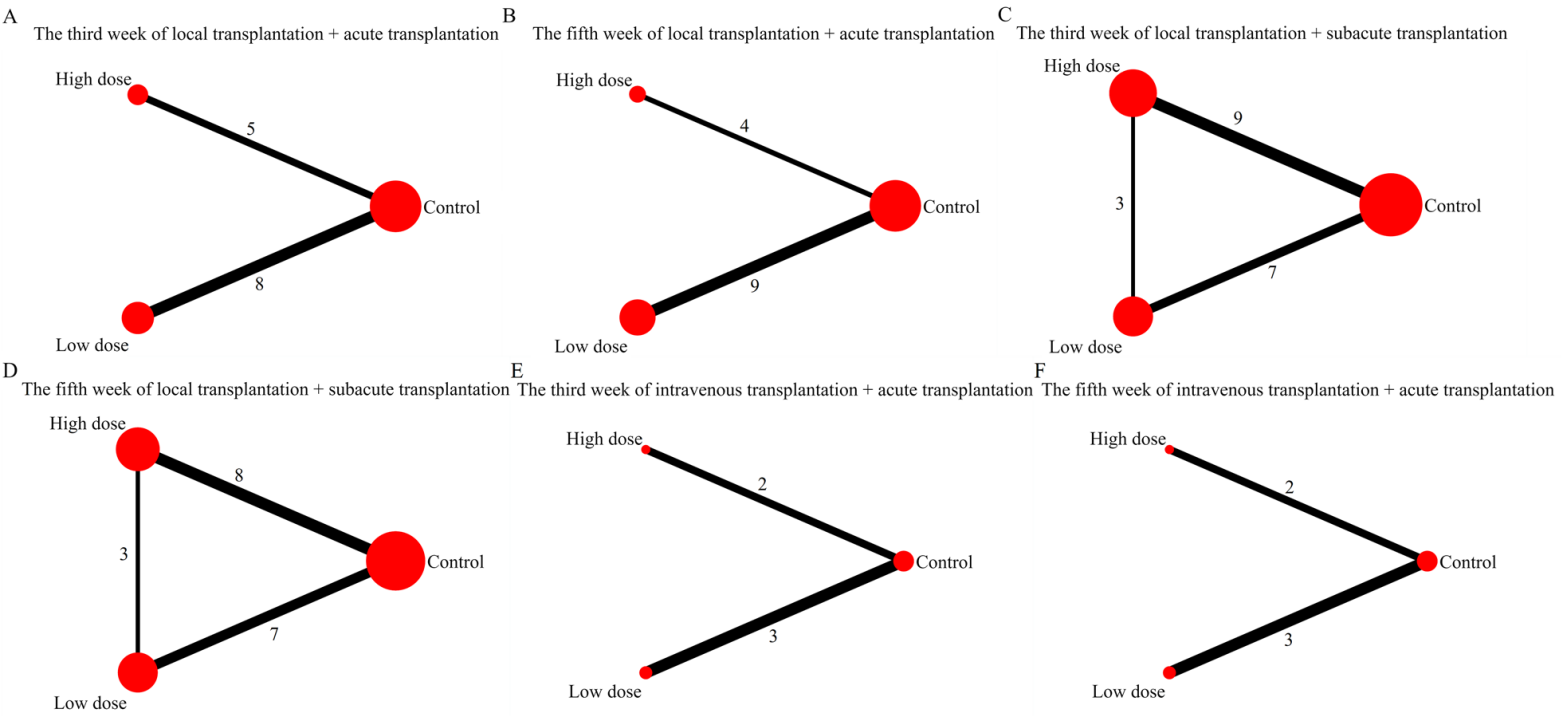
Evidence map for optimal transplantation dose

**Fig. 4:**
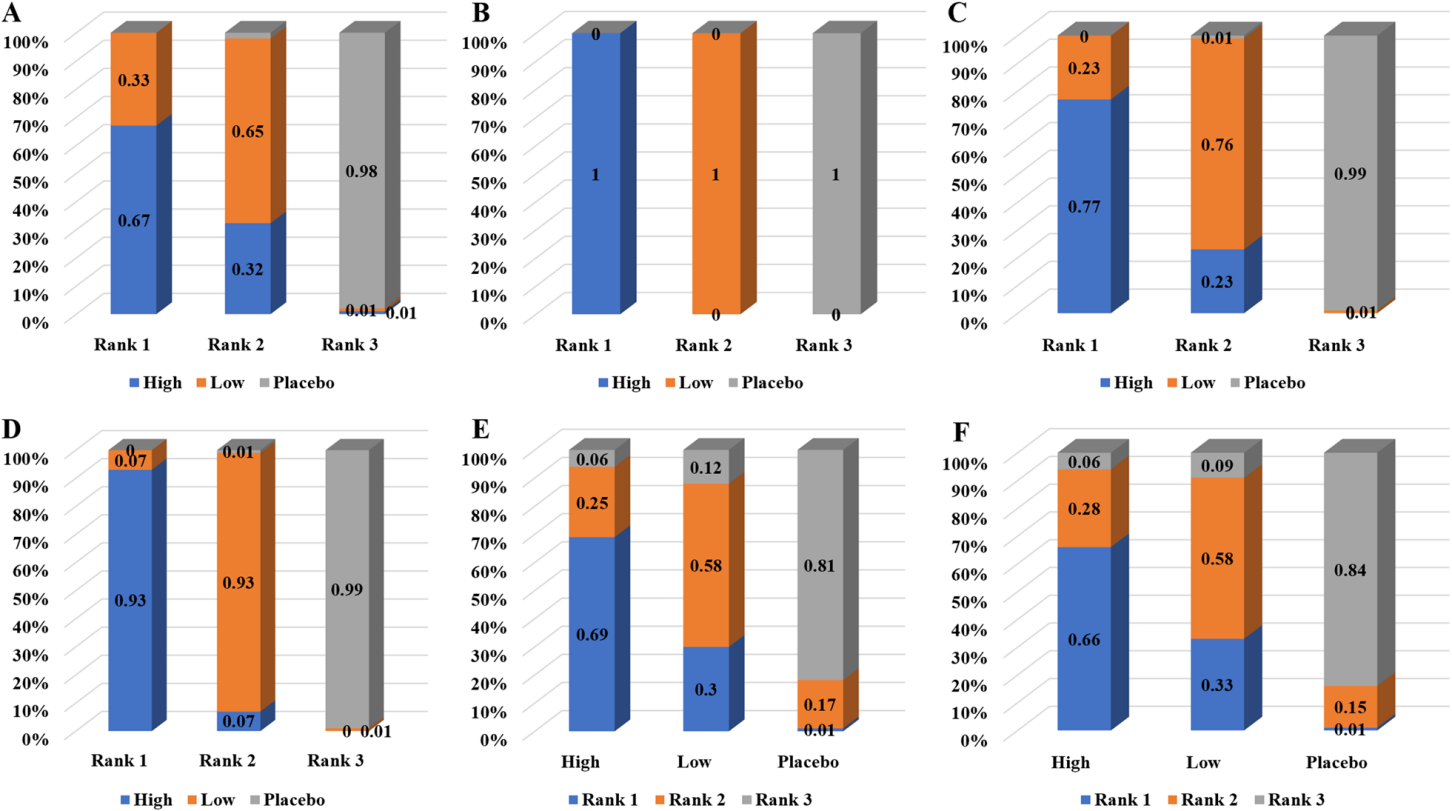
Ranking results of optimal transplantation dose. **A** The third week of local transplantation + acute transplantation. **B** The fifth week of local transplantation + acute transplantation. **C** The third week of local transplantation + subacute transplantation. **D** The fifth week of local transplantation + subacute transplantation. E. The third week of intravenous transplantation + acute transplantation. **F** The fifth week of intravenous transplantation + acute transplantation.

**Fig. 5:**
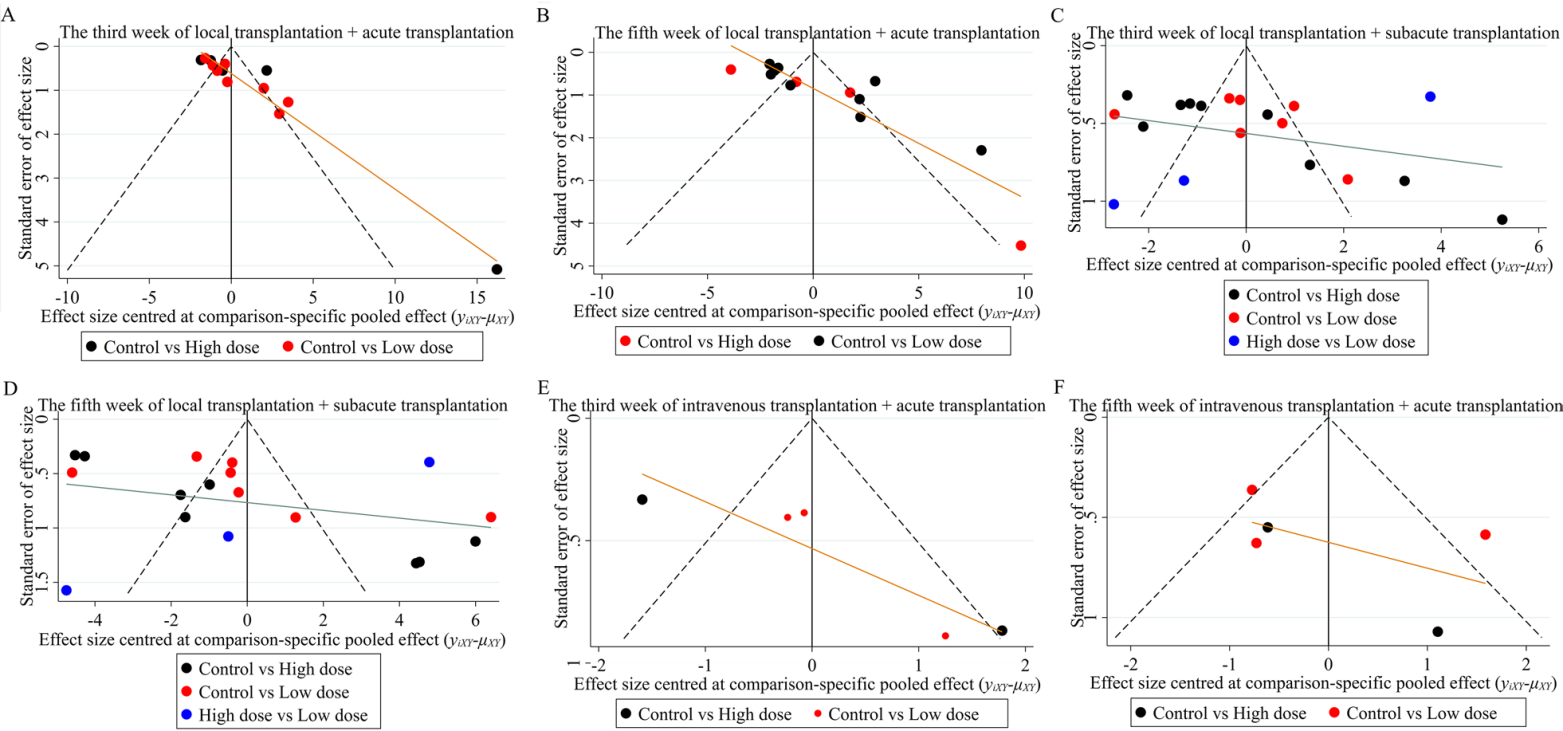
Comparison-corrected funnel plots of optimal transplantation dose

The evidence map showed that only 3 studies have investigated the effect of different transplantation doses of UCMSCs (Fig. 3). The results of network meta-analysis showed that in the fifth week of the local transplantation + acute phase transplantation group, the effect of high-dose transplantation was significantly better than that of low-dose transplantation; other than that, the differences between the other groups were not statistically significant (Table 3). At this time, the ranking results all showed that the effect of high-dose stem cell transplantation may be better than that of low-dose stem cell transplantation (Fig. 4). Asymmetric comparison-corrected funnel plots indicated possible publication bias and small sample effects (Fig. 5).

**Table 3:** Network meta-analysis results of optimal transplantation dose

Group	The third week (SMD)	The fifth week (SMD)
Local transplantation + acute transplantation	0.38 (− 1.57, 2.31)	2.41 (0.92, 3.86)
Local transplantation + subacute transplantation	0.59 (− 1.14, 2.25)	1.58 (− 0.59, 3.76)
Intravenous transplantation + acute transplantation	1.17 (− 4.73, 6.94)	1.50 (− 6.89, 9.86)

#### The results of traditional meta-analysis of optimal transplantation route (minor SCI)

When exploring the optimal route of stem cell transplantation, we divided the included studies into four subgroups: acute phase + high dose transplantation group; acute phase + low dose transplantation group; subacute phase + high dose transplantation group; and subacute phase + low dose transplantation group. Since only local transplantation was performed in the subacute phase + low dose transplantation group, the transplantation effect was not compared with that of intravenous transplantation. The results of traditional meta-analysis at different time points in the other three groups all showed that stem cells can significantly improve the motor function of rats with SCI, and the therapeutic effect of local transplantation is better than that of intravenous transplantation. See Table 4 for details.

**Table 4:** Traditional meta-analysis results of optimal transplantation route

Group	Transplantation route	The third week	The fifth week
Acute phase + high dose transplantation	Local	2.93 (0.90, 4.96)	6.28 (2.96, 9.81)
	Intravenous	0.99 (0.45, 1.52)	4.63 (2.83, 6.43)
Acute phase + low dose transplantation	Local	2.22 (1.23, 3.22)	2.83 (1.56, 4.11)
	Intravenous	1.96 (0.36, 3.56)	1.80 (0.30, 3.30)
Subacute phase + high dose transplantation	Local	2.56 (1.50, 3.62)	3.57 (2.40, 4.73)
	Intravenous	1.70 (0.31, 3.09)	1.87 (0.54, 3.20)

#### The results of network meta-analysis of optimal transplantation route (minor SCI)

The evidence map showed that only one study has investigated the effect of different transplantation routes of UCMSCs (Additional file 1: Fig. S1). The results of network meta-analysis at different time points in different groups all showed that there was no significant difference in the therapeutic effect of different transplantation routes groups (Table 5). At this time, the ranking results all showed that the effect of local transplantation may be better than that of intravenous transplantation (Additional file 1: Fig. S2). Asymmetric comparison-corrected funnel plots indicated possible publication bias and small sample effects (Additional file 1: Fig. S3).

**Table 5:** Network meta-analysis results of optimal transplantation route

Group	The third week (SMD)	The fifth week (SMD)
Acute phase + high dose transplantation	− 0.84 (− 5.29, 3.44)	− 0.49 (− 5.75, 5.01)
Acute phase + low dose transplantation	0.06 (− 1.26, 1.41)	− 1.06 (− 3.56, 1.39)
Subacute phase + high dose transplantation	2.14 (− 0.90, 5.40)	1.53 (− 1.73, 4.80)

#### The results of traditional meta-analysis of optimal transplantation timing (minor SCI)

When exploring the optimal timing of stem cell transplantation, we divided the included studies into four subgroups: high dose + local transplantation group; low dose + local transplantation group; high dose + intravenous transplantation group; and low dose + intravenous transplantation group. Since only acute phase transplantation was performed in the low dose + intravenous transplantation group, the transplantation effect was not compared with that of subacute or chronic phase transplantation. The results of traditional meta-analysis at different time points in the other three groups all showed that stem cells can significantly improve the motor function of rats with SCI, and the therapeutic effect of subacute phase transplantation is better than that of acute phase transplantation (Table 6).

**Table 6:** Traditional meta-analysis results of optimal transplantation timing

Group	Transplantation timing	The third week (SMD)	The fifth week (SMD)
High dose + local transplantation	Acute phase	2.62 (0.31, 4.94)	3.59 (1.99, 7.20)
	Subacute phase	3.29 (2.11, 4.77)	5.41 (3.48, 7.33)
Low dose + local transplantation	Acute phase	2.22 (1.23, 3.22)	2.83 (1.56, 4.11)
	Subacute phase	2.49 (0.48, 3.51)	4.33 (2.53, 6.13)
High dose + intravenous transplantation	Acute phase	1.57 (0.90, 3.03)	1.63 (0.83, 3.43)
	Subacute phase	2.51 (0.81, 3.22)	2.93 (2.11, 3.75)

#### The results of network meta-analysis of optimal transplantation timing (minor SCI)

The evidence map showed that there were no studies investigating the effects of different timings of UCMSCs (Additional file 1: Fig. S4). The results of network meta-analysis showed that in the fifth week of the local transplantation + low dose transplantation group, the effect of subacute transplantation was significantly better than that of acute transplantation; other than that, the differences between the other groups were not statistically significant (Table 7). At this time, the ranking results all showed that the effect of subacute transplantation may be better than that of acute transplantation (Additional file 1: Fig. S5). Asymmetric comparison-corrected funnel plots indicated possible publication bias and small sample effects (Additional file 1: Fig. S6).

**Table 7:** Network meta-analysis results of optimal transplantation timing

Group	The third week (SMD)	The fifth week (SMD)
High dose + local transplantation	0.04 (− 2.29, 2.30)	− 0.04 (− 2.55, 2.52)
Low dose + local transplantation	− 0.42 (− 1.84, 0.99)	2.54 (0.35, 4.79)
High dose + intravenous transplantation	− 0.62 (− 7.70, 6.12)	1.59 (− 5.28, 8.79)

#### Sensitivity analysis

**Fig. 6:**
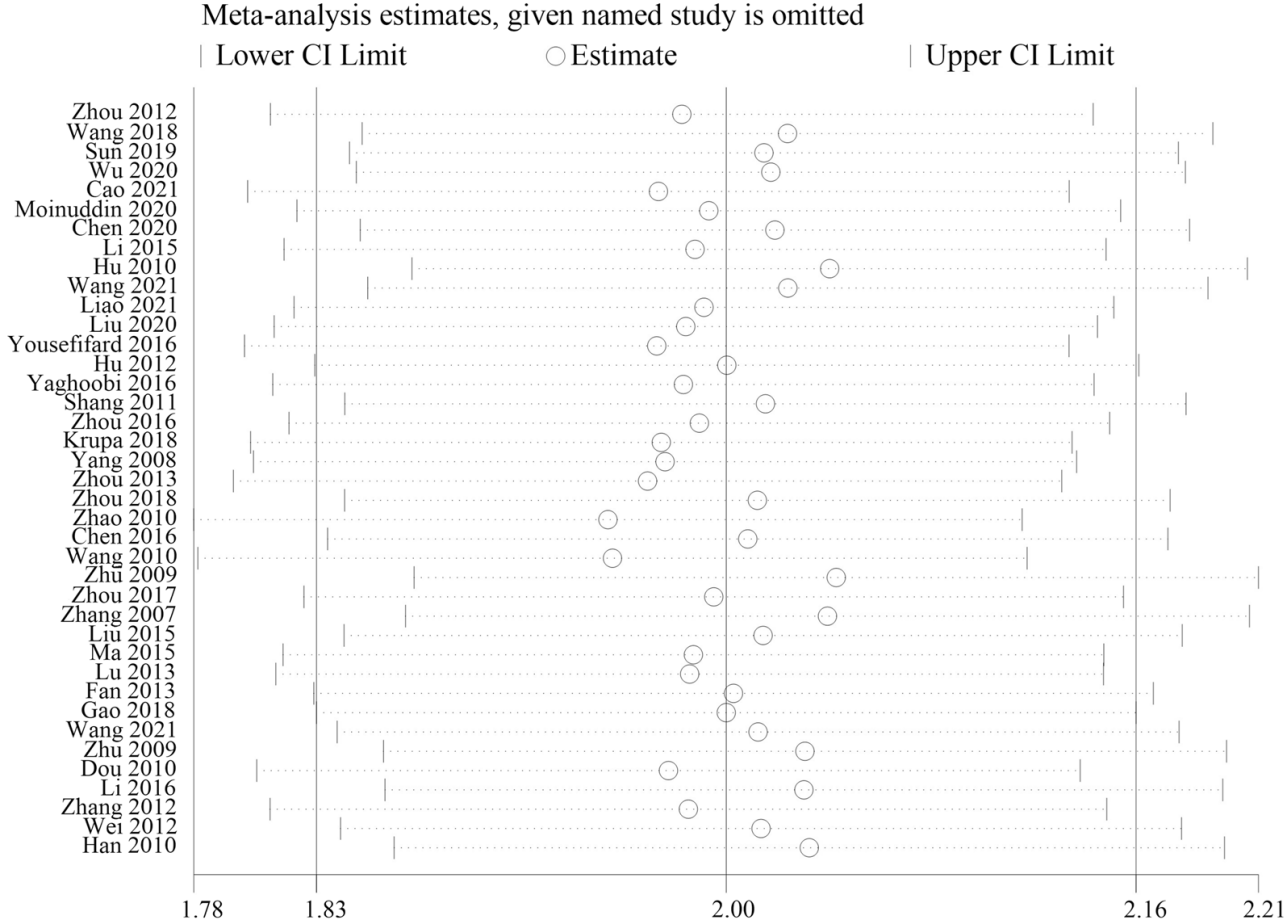
Sensitivity analysis results

The large heterogeneity of UCMSCs in different studies, coupled with differences in operating procedures, makes it difficult to standardize the quality of UCMSCs. To ensure the reliability of the meta-analysis results, we performed a sensitivity analysis on the data of the third week of included studies to explore the impact of different studies on the stability of the meta-analysis results. The results of sensitivity analysis showed that the included studies had good consistency (Fig. 6).

## Discussion

### Evidence summary

The transplantation dose of stem cells determines the number of effective stem cells reaching the injured site. Compared with bone marrow mesenchymal stem cells, UCMSCs are smaller in size, more mobile in the host environment, easier to pass through various barriers in the body, and more cells can be accommodated in a smaller volume. Therefore, the dose-dependent effect of UCMSCs is more obvious [25]. However, excessive transplantation dose may lead to the formation of cell mass, resulting in embolism of organs and tissues, thereby affecting the function of stem cells. For example, by comparing the therapeutic effects of different doses of stem cells, Won et al. found that the therapeutic effect of stem cell transplantation dose of 1 × 10^6^ was significantly better than that of 3 × 10^5^ and 3 × 10^6^ [26]. This is because an excessively high dose of stem cell suspension is relatively viscous and forms cell clumps, resulting in a greatly reduced number of effective stem cells migrating to the site of injury. On the contrary, too low dose of stem cells cannot fully exert the repair effect because there is not enough dose of stem cells to reach the damaged site. For example, the study by Paul et al. found that mesenchymal stem cells engraftment was only reported as 2.3% and 1.6%, after 4 and 21 days, respectively [27]. The transplantation dose of stem cells had to be increased to ensure sufficient stem cells play a repairing role. Therefore, to explore the optimal dose of stem cell transplantation is very important to improve the therapeutic effect of stem cells. Through the traditional meta-analysis and network meta-analysis, we found that no matter what kind of transplantation route and timing, the same results were obtained, that is, high dose (≥ 1 × 10^6^) of stem cell transplantation is better than low dose (< 1 × 10^6^) of stem cell transplantation. This is consistent with the results of Shang et al. Their research based on adipose mesenchymal stem cells also found that high-dose (≥ 1 × 10^6^) stem cell transplantation was more effective than low-dose stem cell transplantation [28]. In addition, studies have shown that too high dose of cell transplantation can trigger a strong inflammatory response, thus reducing the repair effect of stem cells [29]. However, since the transplantation dose of stem cells in most of our studies is about 1 × 10^6^ , and the number of studies included is limited, it is impossible to explore a more accurate stem cell transplantation dose through further meta-analysis. Therefore, more research is needed in the future to explore the optimal dose of stem cells for transplantation. Interestingly, among the studies we included, one study compared treatment effects between repeat and single transplantation [30]. It was found that when the same dose of UCMSCs was transplanted, the effect of repeated transplantation was significantly better than that of single transplantation. Indeed, due to the low survival rate of exogenous cells, repeated transplantation can lead to higher cell survival efficiency and increase the duration of cell nutrition and immunomodulatory effects. As the cell therapy of spinal cord injury is still in the experimental stage, the specific repair mechanism of stem cells is still unclear, and there is no accurate explanation of why repeated transplantation is better than single transplantation. However, as a potential strategy to improve the repair effect of stem cells, repeated transplantation is also a direction to be paid attention to in the future.

At present, local and intravascular transplantation are the main routes of cell therapy for spinal cord injury [31]. Local transplantation is the direct injection of cells into the parenchyma of the spinal cord or intrathecal transplantation to the subarachnoid space. Endovascular transplantation usually includes both arterial and venous routes, with the venous route being the most used. However, in most studies of intravenously transplanted stem cells, the cells did not migrate to the site of spinal cord injury, but to the lungs, spleen, and kidneys [31]. For example, studies have shown that about 96% of stem cells by intravenous transplantation are trapped in the lungs and do not reach the site of spinal cord injury [32]. In addition, many animals in the intravenous group died of pulmonary embolism shortly after transplantation [31]. There are also studies comparing the efficiency of different routes of transplanting stem cells. The results showed that local transplantation of stem cells had the highest efficiency; however, about 6 weeks after transplantation, the level of cells at the site of the spinal cord injury dropped to about 10% of their original level. [33, 34]. In the intravenous transplantation group, no transplanted stem cells were found at the injury site at all, and all cells accumulated in the chest of the mice, which may lead to further pulmonary embolism [35]. This is consistent with our meta-analysis, and our study also found that the therapeutic effect of local stem cell transplantation is significantly better than that of intravenous transplantation. However, the disadvantages of local transplantation cannot be ignored. For example, local transplantation may cause secondary injury due to invasive operations such as laminectomy and needle insertion. In addition, the uncontrollable dose, speed, and pressure of transplantation may further aggravate spinal cord injury. Therefore, future animal studies need to further optimize the specific administration steps of local transplantation in order to reduce the incidence of secondary injury.

After spinal cord injury, the rapidly activated inflammatory response in animals induces a cytotoxic environment that affects stem cell survival and differentiation, while the infiltration of neutrophils and microglia/macrophages further exacerbates the degree of inflammation [36, 37]. Therefore, the acute phase is not the optimal time for stem cell transplantation. In contrast, in the chronic phase, the injury signal generated at the spinal cord injury site is gradually lost, making it difficult to attract exogenous stem cells to migrate to the injury site. In addition, many glial scars are formed at the injured site, which hinders nerve regeneration. Therefore, the effect of stem cell transplantation in the chronic phase may also be poor [38]. The results of Ann et al. support the conclusion that the effect of stem cell transplantation in acute and chronic spinal cord injury is worse than that in subacute spinal cord injury [39]. This is consistent with our findings. Both our traditional meta-analysis and network meta-analysis showed that the effect of stem cell transplantation in the subacute phase was better than that in the acute phase. However, few studies have explored the effect of stem cell transplantation in the chronic phase, so we have not concluded that stem cell transplantation in the subacute phase is superior to that in the chronic phase. Therefore, more studies are needed to explore the therapeutic effects of stem cells in different transplantation periods in order to improve the survival rate of stem cells in animals with spinal cord injury.

In summary, our study found that a higher transplantation dose (≥ 1 × 10^6^), local transplantation, and subacute transplantation are the optimal transplantation strategies of UCNSCs. Obviously, the therapeutic effect of stem cells is comprehensively affected by the dose, route, and timing of transplantation. Therefore, more animal experiments, especially direct comparison studies, are needed in the future to further explore the optimal stem cell transplantation strategy to improve the therapeutic effect of stem cells.

### Internal validity

Internal validity refers to the degree to which the research results are consistent with the real situation of the actual research objects, and to answer whether a study itself is true or effective. We assessed the inherent risk of bias of included studies by SYRCLE's risk of bias tool to reflect the internal validity of the current study.

1. Selection bias: Choices made by researchers in the design, conduct, and interpretation of experiments can introduce selection bias that can lead to false-positive results. Randomization, concealment, and balancing the baseline characteristics of animals are important means of reducing selection bias [40]. Although the baseline characteristics of 95% of the animals in the study are balanced, and all studies are randomized controlled trials, only 5% of the studies have reported specific random grouping methods, and there have been no studies on the implementation of covert grouping, resulting in certain selection bias in the included studies.
2. Implementation bias: The implementation bias in animal experiments mainly refers to whether to implement random placement of animals and blind method for animal breeders and researchers. Although 60% of the studies randomly housed experimental animals during the experiment, all included studies were unable to judge whether animal breeders and/or researchers were blinded, resulting in a certain implementation bias in the included studies.
3. Measurement bias: The evaluator of the results inevitably expects the animals in the experimental group to show positive results, and thus inadvertently overestimates the effect of the treatment in the experimental group of animals. If the blind method is not applied to the evaluator of the results, the existence of measurement bias increases the probability of positive results or even false-positive results [41]. Although 70% of the studies blinded the evaluators of the results, none reported the specific blinding process. Therefore, future research needs to pay more attention to the application of blinding in experiments, and at the same time, provide more experimental details to reduce various potential biases.
4. Reporting bias: Animal models are valuable for developing effective treatments, provided their experiments are carefully designed, interpreted, and reported. Inadequate reporting of experimental results can lead to findings that are uninterpretable and difficult to replicate. Based on a comprehensive judgment of the purposes, experimental methods, and outcome measures of the included studies, although all the 40 included studies clearly reported all the expected results, none of them had access to the original protocols for their studies and could not ultimately determine whether all their results were reported unbiasedly in accordance with the protocols. Therefore, we suggest prospective registration of animal studies to improve the quality of animal experiments [42].
5. Publication bias: The publication rate of animal studies is only 60–67%, especially studies with negative results remain unpublished [43, 44]. If the negative results are not included in the systematic review, the effect of intervention will be overestimated. The asymmetric comparative correction funnel plot of this study shows that there is a certain publication bias in the current field. Therefore, it is necessary to formulate relevant policies to encourage and require journals to publish negative or neutral results in order to avoid the “drawer document” effect and reduce the impact of publication bias on its results [45].

### External validity

External validity refers to the degree of consistency between the research results and the real situation of the inference object, mainly to answer whether research can be applied to people outside the research object. In animal studies, several aspects of external validity should be considered when translating experimental results into clinical trials.

1. Nerve regeneration treatments in animal experiments have mainly focused on the acute and subacute phases, when salvageable nerve cells still exist and glial scarring is not yet fully formed. Unfortunately, 95% of patients are in the chronic phase of the injury [46].
2. Patients with cervical spinal cord injury account for about 60% of spinal cord injuries. However, most basic research uses animal models of thoracic spinal cord injury [45], which reduces the inspiration of animal experimental results for clinical research [47].
3. Due to the increasing incidence of spinal cord injury in the elderly, the co-diseases of patients, such as diabetes and hypertension, will reduce the proliferation and differentiation potential of stem cells and lead to vascular injury. However, animal experiments are difficult to simulate a variety of clinical conditions [46, 48].
4. Longer follow-up results can predict the motor function recovery trajectory of SCI animals more comprehensively, which can reduce the number of subjects required for subsequent clinical trials and better guide clinical practice. However, few preclinical studies have extended the follow-up time to two months after cell transplantation.
5. Due to the insurmountable species differences between experimental animals and humans, it is still doubtful whether the best treatment strategy of stem cells in animal experiments is applicable to humans. For example, the spinal cord injury in rats reaches the plateau in about 5 weeks, while the plateau in patients usually takes more than 6 months [20].

### Strengths and limitations of our study

Key strengths of our study: (1) As the first study in the current field, we systematically evaluated the effect of UCMSCs in the treatment of spinal cord injury and pointed out the problems and improvement direction of current research. (2) In the absence of a direct comparison of evidence, we combine the traditional meta-analysis of subgroup analysis and network meta-analysis to fully prove the best stem cell transplantation strategy from two aspects. (3) We explored the therapeutic effect of UCMSCs at different time points and more comprehensively explored the whole therapeutic effect of stem cells. (4) We assessed the inherent risk of bias of the included studies based on the SYRCLE bias risk assessment tool, and explored the internal and external validity of the current animal experiments, fully demonstrating the feasibility of the current animal experimental research results to be translated into clinical practice.

This systematic review has several limitations: (1) We selected data based on the recovery of motor function in spinal cord-injured rats. Although there is a certain basis, the reliability of this method still needs further research. (2) We only included the BBB score, which is widely used and best reflects the effect of stem cell therapy, and did not analyze additional outcome measures. (3) We cannot accurately identify the source of heterogeneity, so we use the random effect model for merger analysis, resulting in our conclusions being more conservative. (4) Searching only Chinese and English databases may lead to some language bias. (5) Failure to search gray literature and conference abstracts may lead to publication bias.

## Conclusion

The systematic review of animal experiments can fully explore the therapeutic effect of intervention measures and point out the problems and limitations of the current research. Based on the systematic reviews of 40 animal studies, we found that in the subacute stage, local transplantation of higher doses of umbilical cord mesenchymal stem cells can significantly improve the effect of repairing spinal cord injury. However, the current animal studies have some problems in the design, implementation, measurement, and reporting of the results, which leads to many potential bias risks. In addition, the internal and external validity of the animal experimental results are limited, which reduces the reliability of the experimental results and the guidance and enlightening significance for future research to a certain extent. Therefore, future animal experiments need to be carefully and scientifically designed and implemented, and the optimal treatment strategy of umbilical cord mesenchymal stem cells should be explored more comprehensively.

## Supplementary Information

**Additional file1: Table S1**: Chinese and English search strategies. **Table S2**: Basic information included in the study. **Figure S1**: Evidence map for optimal transplantation route. **Figure S2**: Ranking results of optimal transplantation route. (A. The third week of acute phase + high dose transplantation. B. The fifth week of acute phase + high dose transplantation. C. The third week of acute phase + low dose transplantation. D. The fifth week of acute phase + low dose transplantation. E. The third week of subacute phase + high dose transplantation. F. The fifth week of subacute phase + high dose transplantation). **Figure S3**: Comparison-corrected funnel plots of optimal transplantation dose. **Figure S4**: Evidence map for optimal transplantation timing. **Figure S5**: Ranking results of optimal transplantation timing. (A. The third week of high dose + local transplantation. B. The fifth week of high dose + local transplantation. C. The third week of low dose + local transplantation. D. The fifth week of low dose + local transplantation. E. The third week of high dose + intravenous transplantation. F. The fifth week of high dose + intravenous transplantation). **Figure S6**: Comparison-corrected funnel plots of optimal transplantation dose.

## Data Availability

The datasets used and/or analyzed during the current study are available from the corresponding author on reasonable request.

## Abbreviations

UCMSCs: Umbilical cord mesenchymal stem cells
SCI: Spinal cord injury
BBB: Basso–Beattie–Bresnahan
SMD: Standardized mean difference
