# Supplementary material for: The optimal transplantation strategy of umbilical cord mesenchymal stem cells in spinal cord injury: a systematic review and network meta-analysis based on animal studies
Source: Stem Cell Res Ther. 2022 Sep 2;13:441. doi: 10.1186/s13287-022-03103-8 (PMC9438219; doi:10.1186/s13287-022-03103-8)
Supplement: Supplementary file 1 — Additional file1: Table S1: Chinese and English search strategies. Table S2: Basic information included in the study. Figure S1: Evidence map for optimal transplantation route. Figure S2: Ranking results of optimal transplantation route. (A. The third week of acute phase + high dose transplantation. B. The fifth week of acute phase + high dose transplantation. C. The third week of acute phase + low dose transplantation. D. The fifth week of acute phase + low dose transplantation. E. The third week of subacute phase + high dose transplantation. F. The fifth week of subacute phase + high dose transplantation). Figure S3: Comparison-corrected funnel plots of optimal transplantation dose. Figure S4: Evidence map for optimal transplantation timing. Figure S5: Ranking results of optimal transplantation timing. (A. The third week of high dose + local transplantation. B. The fifth week of high dose + local transplantation. C. The third week of low dose + local transplantation. D. The fifth week of low dose + local transplantation. E. The third week of high dose + intravenous transplantation. F. The fifth week of high dose + intravenous transplantation). Figure S6: Comparison-corrected funnel plots of optimal transplantation dose. [file 13287_2022_3103_MOESM1_ESM.docx]

**The optimal transplantation strategy of umbilical cord mesenchymal stem cells in spinal cord injury: A systematic review and meta-analysis based on animal studies**

**Supplementary Material Table 1: Chinese and English search strategies**

| PubMed  #1: "Spinal Cord Injuries"[Mesh] 52,542  #2: "Spinal cord injury"[Title/Abstract] OR "Spinal injury"[Title/Abstract] OR "Spinal Cord Trauma"[Title/Abstract] OR "Spinal Cord Transection"[Title/Abstract] OR "Spinal Cord Laceration"[Title/Abstract] OR "Post-Traumatic Myelopathy"[Title/Abstract] OR "Spinal Cord Contusion"[Title/Abstract] 42,916  #3: #1 OR #2 66,485  #4: ("Umbilical Cord"[Mesh]) AND "Stem Cells"[Mesh] 2,241  #5: "umbilical cord mesenchymal stem cells"[Title/Abstract] OR "umbilical cord stem cells"[Title/Abstract] OR (umbilical cord[Title/Abstract] AND ("stem cells"[Title/Abstract] OR "stem cell"[Title/Abstract])) OR UCMSCs[Title/Abstract] 7,455  #6: #4 OR #5 8,057  #7: #3 AND #6 150  Web of science  (TS=(“Spinal cord injury” OR “Spinal injury” OR “Spinal Cord Trauma” OR “Spinal Cord Transection” OR “Spinal Cord Laceration” OR “Post-Traumatic Myelopathy” OR “Spinal Cord Contusion”)) AND TS=(“umbilical cord mesenchymal stem cells” OR “umbilical cord stem cells” OR (umbilical cord AND (“stem cells” OR “stem cell”)) OR UCMSCs) 318  Embase  #1: (Spinal cord injury or Spinal injury or Spinal Cord Trauma or Spinal Cord Transection or Spinal Cord Laceration or Post-Traumatic Myelopathy or Spinal Cord Contusion).mp. [mp=title, abstract, heading word, drug trade name, original title, device manufacturer, drug manufacturer, device trade name, keyword heading word, floating subheading word, candidate term word] 65183  #2: *spinal cord injury/ 32346  #3: *spinal cord transection/ 761  #4: 1 or 2 or 3 65310  #5: exp umbilical cord mesenchymal stem cell/ 620  #6: (umbilical cord mesenchymal stem cells or umbilical cord stem cells or (umbilical cord and (stem cells or stem cell)) or UCMSC).mp. [mp=title, abstract, heading word, drug trade name, original title, device manufacturer, drug manufacturer, device trade name, keyword heading word, floating subheading word, candidate term word] 22035  #7: 5 or 6 22035  #8: 4 and 7 312  CNKI/高级检索; CNKI / Advanced Search  主题:脊髓损伤 AND 脐带间充质干细胞 (60)  Subject: spinal cord injury AND umbilical cord mesenchymal stem cells (60)  万方/高级检索; Wanfang database/ Advanced Search  主题:脊髓损伤 AND 脐带间充质干细胞 (77)  Subject: spinal cord injury AND umbilical cord mesenchymal stem cells (77)  VIP/高级检索; VIP database/ Advanced Search  题名或关键词: 脊髓损伤 AND脐带间充质干细胞(52)  Title or keyword: spinal cord injury AND umbilical cord mesenchymal stem cells (52)  CBM/高级检索; CBM / Advanced Search  #1:"脊髓损伤"[不加权:扩展] 61300  #2:"脊髓损伤"[常用字段:智能] 79316  #3: #2 OR #3 79316  #4:"脐带间充质干细胞"[常用字段:智能] 2029  #5: #3 AND #4 60  #1:" Spinal cord injury "[unweighted, extended] 61300  #2:" Spinal cord injury "[ common field: smart] 79316  #3: #2 OR #3 79316  #4:"adipose-derived mesenchymal stem cells "[ common field: smart] 2029  #5: #3 AND #4 60 |
| --- |

**Supplementary Material Table 2: Basic information included in the study**

| **No.** | **Author**  **+**  **Year** | **Country** | **Type of study** | **Species** | **Gender** | **Weight** | **Age** | **Sample size** | **Model types** | **Stem cell transplantation** | | | | **Control** |
| --- | --- | --- | --- | --- | --- | --- | --- | --- | --- | --- | --- | --- | --- | --- |
|  |  |  |  |  |  |  |  |  |  | **Dose** | **Source** | **Route** | **Timing** |  |
| 1 | Zhou 2012[1] | China | RCT | SD rats | Female | 200-250g | / | 8/8 | Contusion | 2×10^5^ | Human cord | Intralesional | 0d | PBS |
| 2 | Wang 2018[2] | China | RCT | SD rats | Female | 174-236g | 8-10 weeks | 28/28 | Compression | 4×10^5^ | Human cord | Intralesional | 3d | Blank |
| 3 | Sun 2019[3] | China | RCT | Wistar rats | Female | 180-2210g | Adult | 25/25 | Contusion | 6×10^5^ | Human cord | Intralesional | 8d | PBS |
| 4 | Wu 2020[4] | China | RCT | SD rats | Female | 230-250g | / | 30/30 | Contusion | 1×10^5^ | Human cord | Intralesional | 0d | DMSO |
| 5 | Cao 2021[5] | China | RCT | SD rats | Female | 250g | 6-8 weeks | 12/12 | Compression | 2.5 ×10^5^;4×10^6^ | Human cord | Intralesional | 7d | PBS |
| 6 | Moinuddin 2020[6] | USA | RCT | SD rats | Female | 270-300g | Adult | 6/6 | Contusion | 2 ×10^6^ | SD rats umbilical cord | Intravenous | 7d | Saline |
| 7 | Chen 2020[7] | China | RCT | SD rats | Male | 215-245g | 12 weeks | 8/8 | Contusion | 1 ×10^6^ | Human cord | Intralesional | 1d | Saline |
| 8 | Li 2015[8] | China | RCT | SD rats | Female | 190-210g | Adult | 27/27 | Hemi-sectioned | 1 ×10^6^ | Human cord | Intravenous | 0d | Blank |
| 9 | Hu 2010[9] | China | RCT | SD rats | Female | 230-270g | / | 12/12 | Contusion | 4×10^5^ | Human cord | Intralesional | 1d | DMEM |
| 10 | Wang 2021[10] | China | RCT | SD rats | Female | 180-220g | 8 weeks | 18/18 | Contusion | 5 ×10^6^ | Human cord | Intralesional | 7d | DMEM |
| 11 | Liao 2021[11] | China | RCT | SD rats | Female | 230±40g | Adult | 18/18 | Contusion | 1×10^5^ | Human cord | Intravenous | 1d | Saline |
| 12 | Liu 2020[12] | China | RCT | SD rats | Female | 250±25g | 10 weeks | 51/39 | Contusion | 5×10^5^ | Human cord | Intralesional | 9d | DMEM |
| 13 | Yousefifard 2016[13] | Iran | RCT | Wistar rats | Male | 140-160g | / | 12/12 | Compression | 1 ×10^6^ | Human cord | Intralesional | 7d | Saline |
| 14 | Hu 2012[14] | China | RCT | SD rats | Female | 230-270g | / | 14/12 | Contusion | 4×10^5^ | Human cord | Intralesional | 1d | DMEM |
| 15 | Yaghoobi 2016[15] | Iran | RCT | SD rats | Female | / | Adult | 8/7 | Contusion | 3×10^5^ | Human cord | Intralesional | 1d | Saline |
| 16 | Shang 2011[16] | China | RCT | SD rats | Female | 174-236g | 8-10 weeks | 20/20 | Compression | 1 ×10^6^ | Human cord | Intralesional | 7d | DMEM |
| 17 | Zhou 2016[17] | China | RCT | SD rats | / | / | / | 18/10 | Contusion | 1×10^5^ | Human cord | Intralesional | 1d | DMEM |
| 18 | Krupa 2018[18] | China | RCT | Wistar rats | Male | 275-305g | 10 weeks | 12/11 | Compression | 5×10^5^;1.5×10^6^ | Human cord | Intralesional | 7d | DMEM |
| 19 | Yang 2008[19] | China | RCT | SD rats | Female | 250-300g | Adult | 13/8 | Transection | 5×10^5^ | Human cord | Intralesional | 7d | DMEM |
| 20 | Zhang 2013[20] | China | RCT | SD rats | Male | / | 7 weeks | 20/12 | Contusion | 5 ×10^6^ | Human cord | Intravenous | 14d | Blank |
| 21 | Zhou 2018[21] | China | RCT | SD rats | Female | 200-250g | Adult | 16/16 | Contusion | 3×10^6^ | Human cord | Intravenous | 6d | DMEM |
| 22 | Zhao 2010[22] | China | RCT | Wistar rats | Female | 220±30g | 8 weeks | 20/20 | Contusion | 1×10^5^;1 ×10^6^ | Human cord | Intralesional | 7d | DMEM |
| 23 | Chen 2016[23] | China | RCT | SD rats | Male | / | 12-14 weeks | 20/20 | Contusion | 1.2×10^6^ | Human cord | Intralesional | 3; 21d | Blank |
| 24 | Wang 2010[24] | China | RCT | Wistar rats | Female | 220±20g | 8 weeks | 20/20 | Contusion | 1 ×10^6^ | Human cord | Intralesional | 3; 7; 21d | Blank |
| 25 | Zhu 2009[25] | China | RCT | Wistar rats | Female | / | Adult | 20/20 | Contusion | 1×10^5^ | Wistar rats umbilical cord | Intralesional | 7d | Blank |
| 26 | Zhou 2017[26] | China | RCT | SD rats | Male | 180-237g | Adult | 20/20 | Contusion | 1 ×10^6^ | Human cord | Intralesional | 0d | Blank |
| 27 | Zhang 2007[27] | China | RCT | SD rats | Female and male | 195-285g | Adult | 20/20 | Compression | 1 ×10^6^ | Human cord | Intravenous | 5d | Blank |
| 28 | Liu 2015[28] | China | RCT | Wistar rats | Male | 200-220g | Adult | 20/15 | Contusion | 6 ×10^3^ | Human cord | Intravenous;Intralesional | 0d | DMEM |
| 29 | Ma 2015[29] | China | RCT | SD rats | Female | 200-220g | / | 27/27 | Hemi-sectioned | 1 ×10^6^ | Human cord | Intravenous | 0d | Saline |
| 30 | Lu 2013[30] | China | RCT | SD rats | Male | 220g | Adult | 16/8 | Hemi-sectioned | 6×10^5^ | Human cord | Intralesional | 0d | PBS |
| 31 | Fan 2013[31] | China | RCT | Wistar rats | Female | 220±20g | 8 weeks | 20/20 | Contusion | 1 ×10^6^ | Human cord | Intralesional | 7d | DMEM |
| 32 | Gao 2018[32] | China | RCT | Wistar rats | Female | 250-300g | / | 18/18 | Contusion | 1.5 ×10^7^ | Human cord | Subarachnoid space | 0d | Blank |
| 33 | Wang 2021[33] | China | RCT | SD rats | Female | 230-250g | / | 21/21 | Contusion | 5×10^6^ | Human cord | Intralesional | 0d | Blank |
| 34 | Zhu 2009[34] | China | RCT | Wistar rats | Female | / | 11 weeks | 20/20 | Contusion | 8×10^6^ | Human cord | Intralesional | 7d | DMEM |
| 35 | Huang 2013[35] | China | RCT | SD rats | Male | 270±20g | Adult | 26/20 | Contusion | 3×10^5^ | Human cord | Intralesional | 0d | DMEM |
| 36 | Dou 2010[36] | China | RCT | SD rats | / | 200g | / | 10/10 | Contusion | 5×10^6^ | Human cord | Intravenous | 3d | DMEM |
| 37 | Li 2016[37] | China | RCT | Wistar rats | Female | 200-250g | / | 15/15 | Contusion | 1 ×10^5^ | Human cord | Intravenous | 6h | DMEM |
| 38 | Zhang 2012[38] | China | RCT | Wistar rats | Female | 250-300g | / | 20/20 | Contusion | 3×10^6^ | Human cord | Intravenous | 4h | DMEM |
| 39 | Wei 2012[39] | China | RCT | Wistar rats | Female | 200-220g | 8 weeks | 20/20 | Contusion | 1 ×10^5^ | Human cord | Intralesional | 7d | DMEM |
| 40 | Han 2010[40] | China | RCT | Wistar rats | Female | 230±10g | 8 weeks | 20/8 | Contusion | 1 ×10^6^ | Human cord | Intralesional | 7d | DMEM |

**
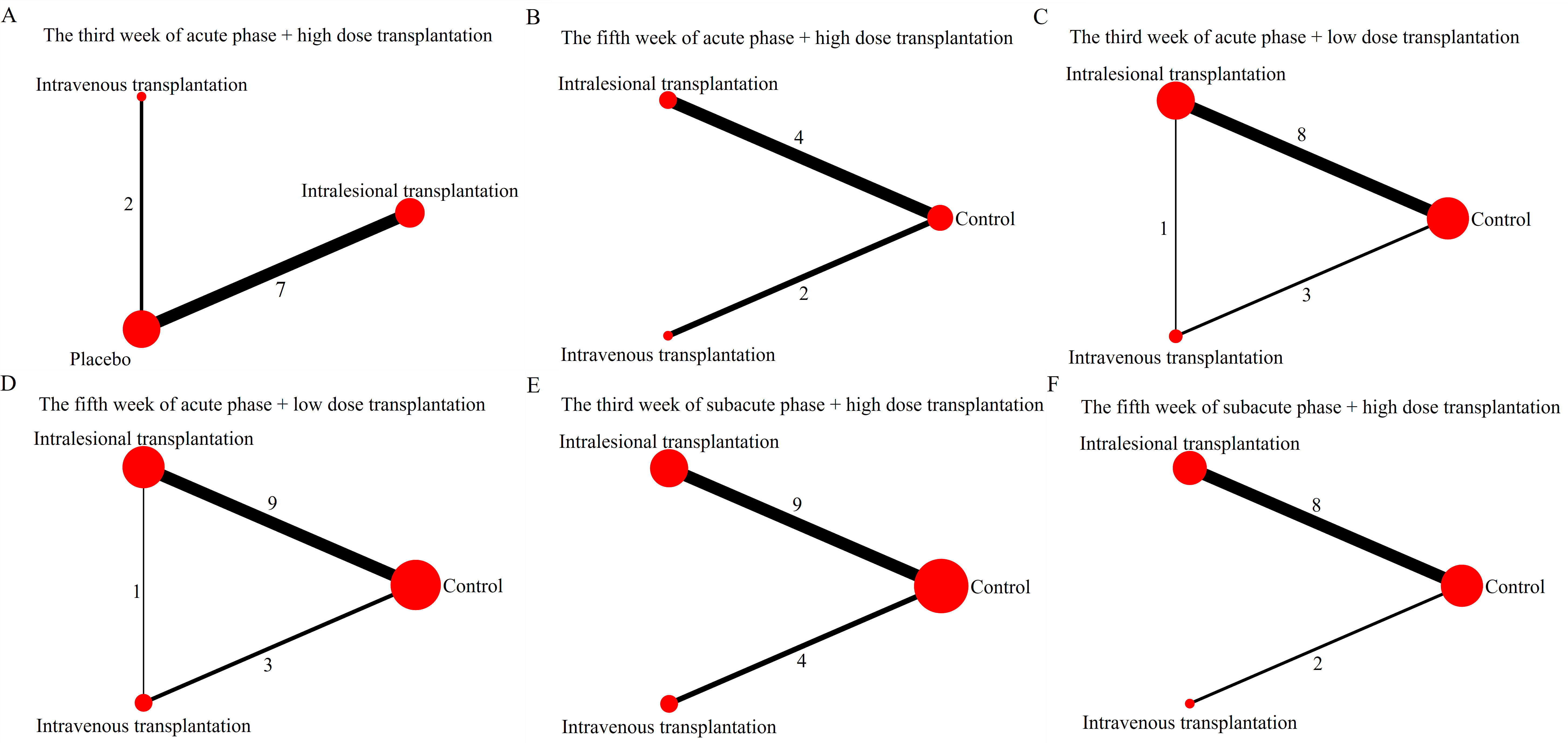
**

**Supplementary Material Figure 1: Evidence map for optimal transplantation route.**

**
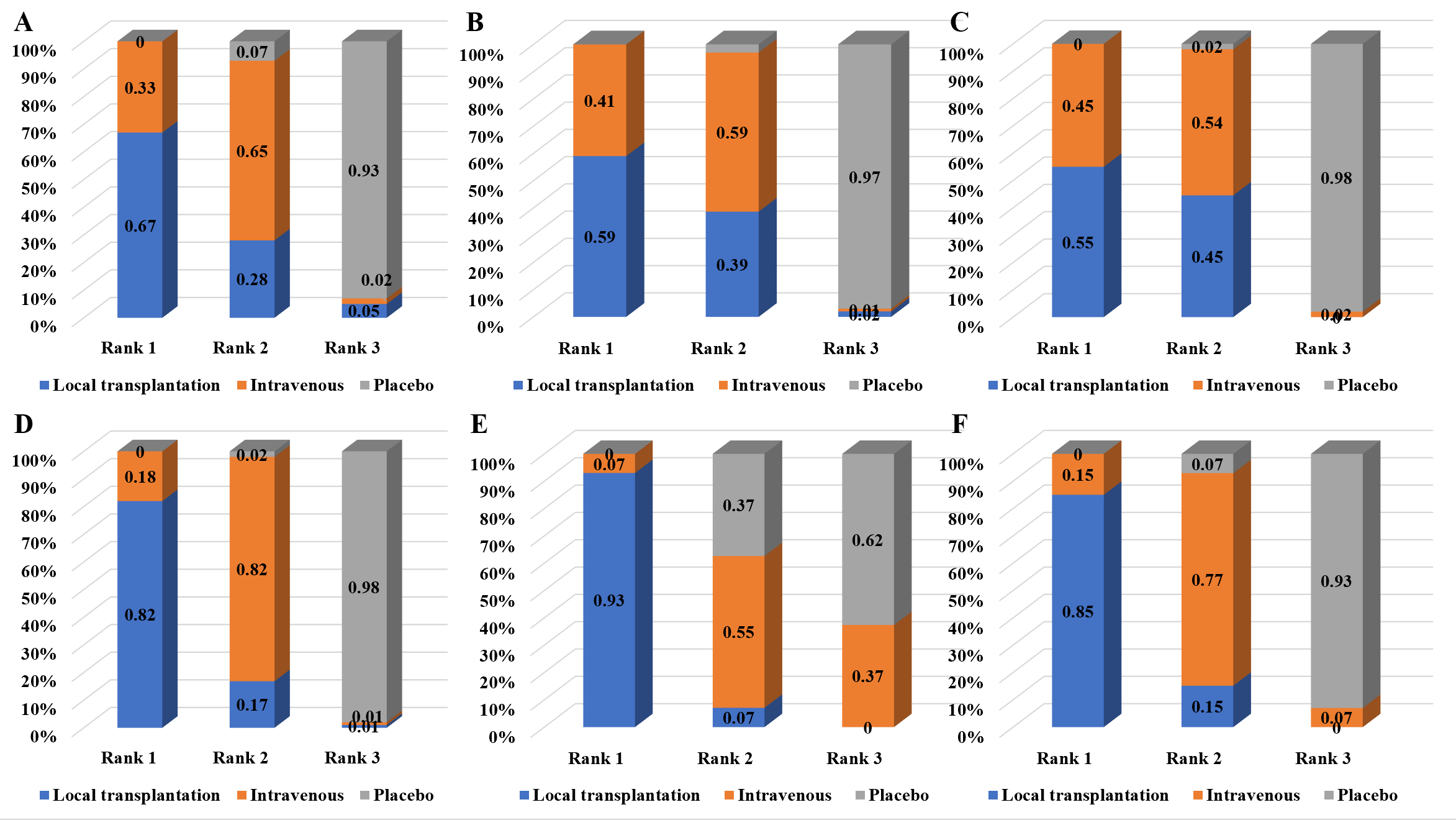
**

**Supplementary Material Figure 2: Ranking results of optimal transplantation route. (A. The third week of acute phase + high dose transplantation. B. The fifth week of acute phase + high dose transplantation. C. The third week of acute phase + low dose transplantation. D. The fifth week of acute phase + low dose transplantation. E. The third week of subacute phase + high dose transplantation. F. The fifth week of subacute phase + high dose transplantation.)**

**
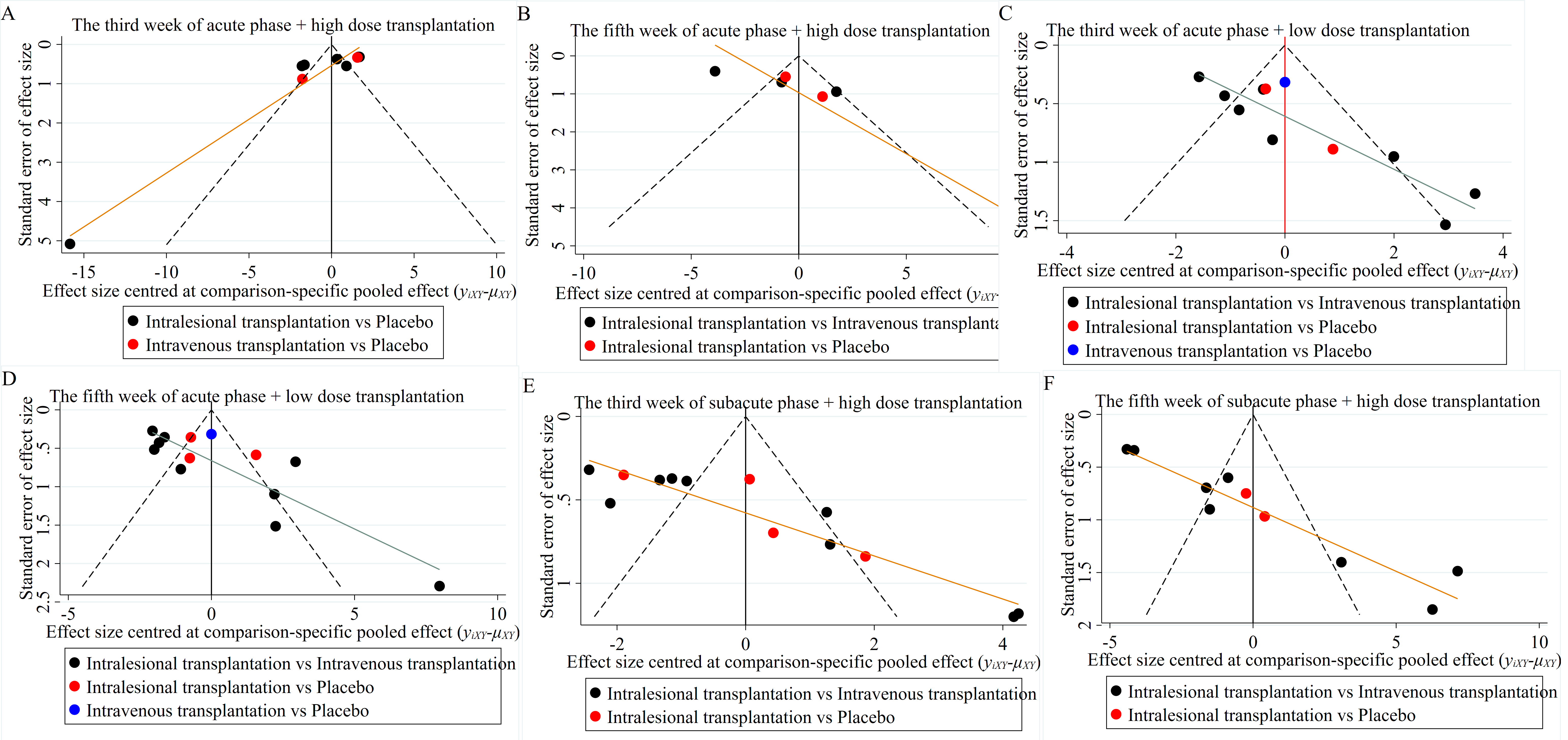
**

**Supplementary Material Figure 3: Comparison-corrected funnel plots of optimal transplantation dose.**

**
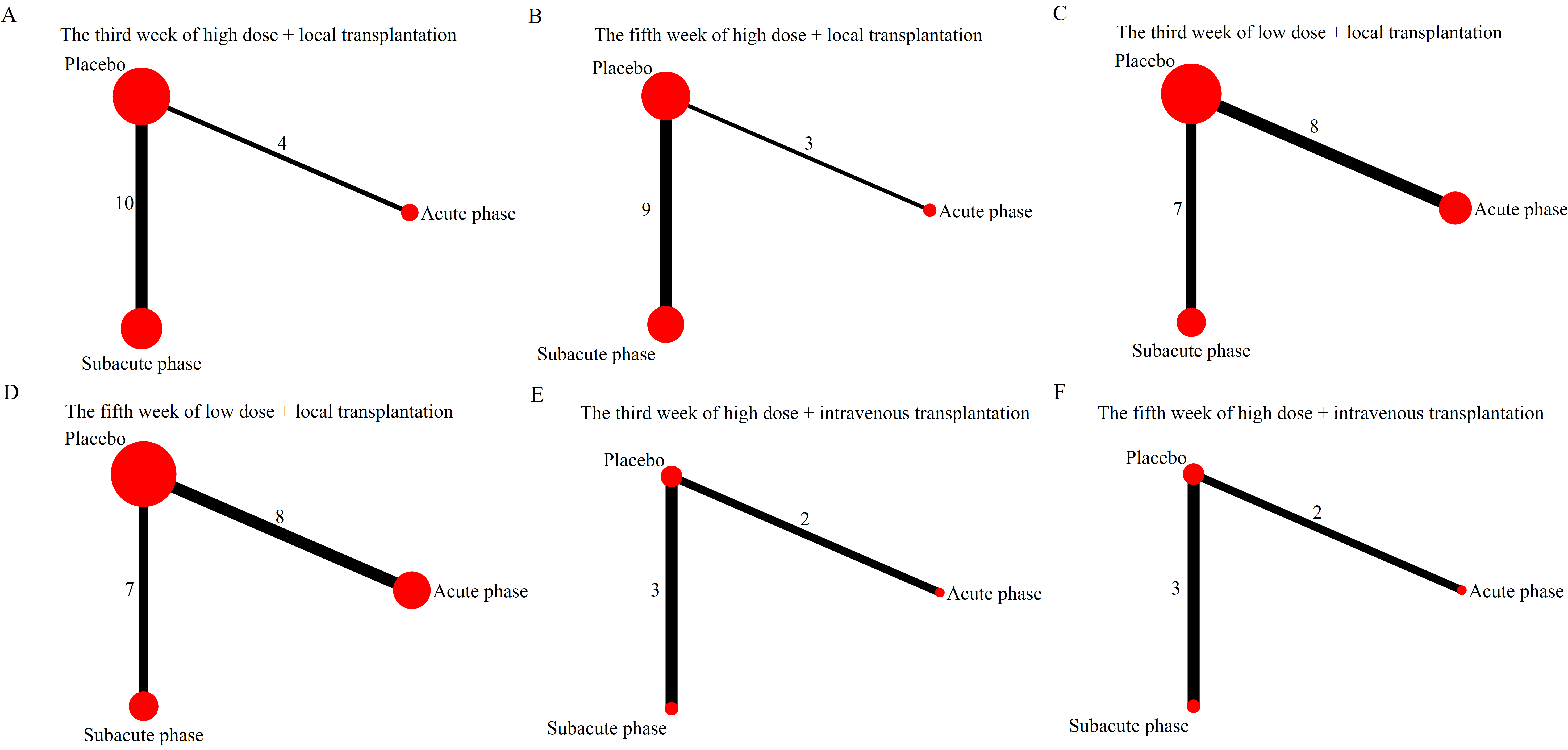
**

**Supplementary Material Figure 4: Evidence map for optimal transplantation timing.**

**
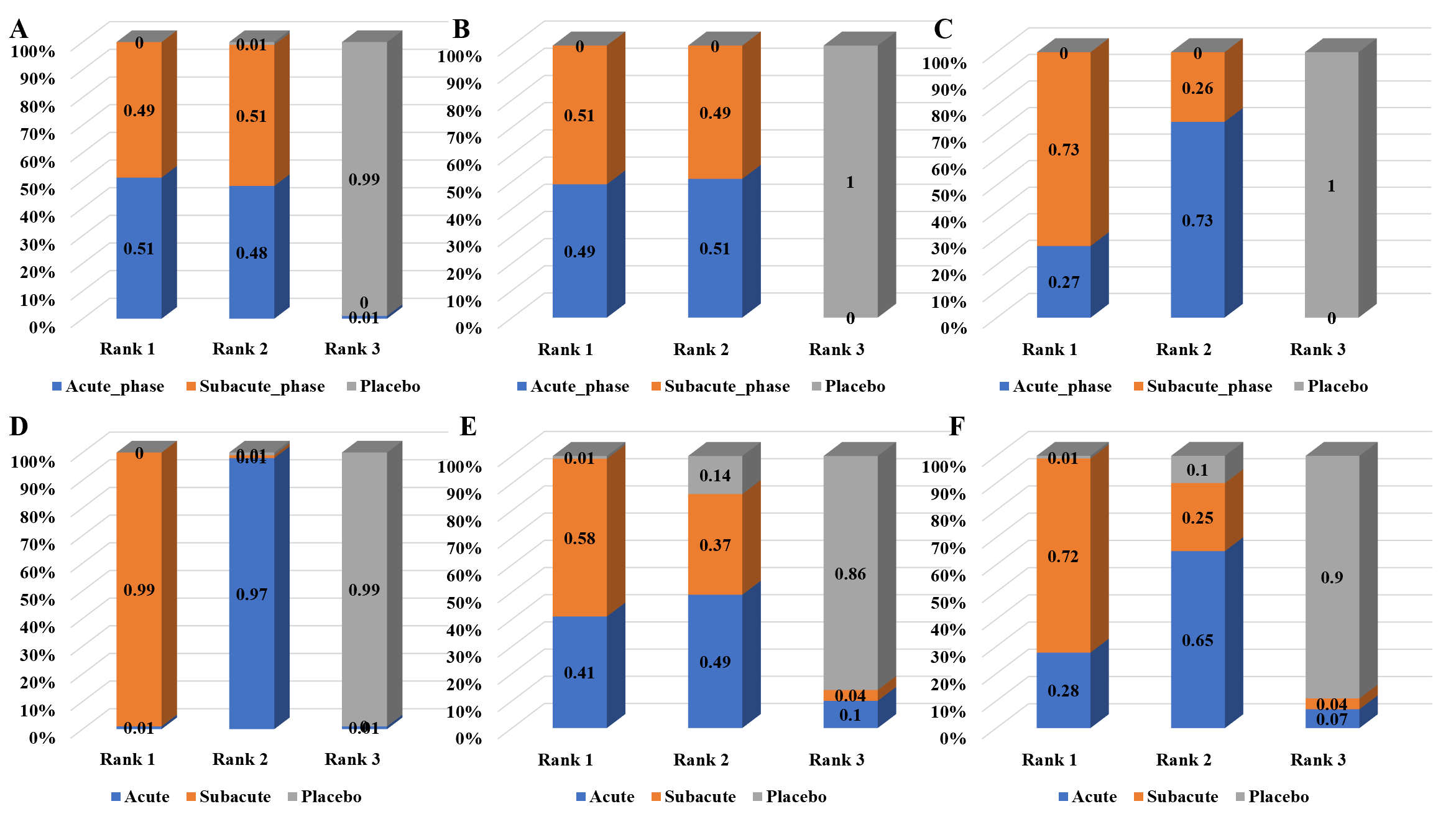
**

**Supplementary Material Figure 5: Ranking results of optimal transplantation timing. (A. The third week of high dose + local transplantation. B. The fifth week of high dose + local transplantation. C. The third week of low dose + local transplantation. D. The fifth week of low dose + local transplantation. E. The third week of high dose + intravenous transplantation. F. The fifth week of high dose + intravenous transplantation.)**

**
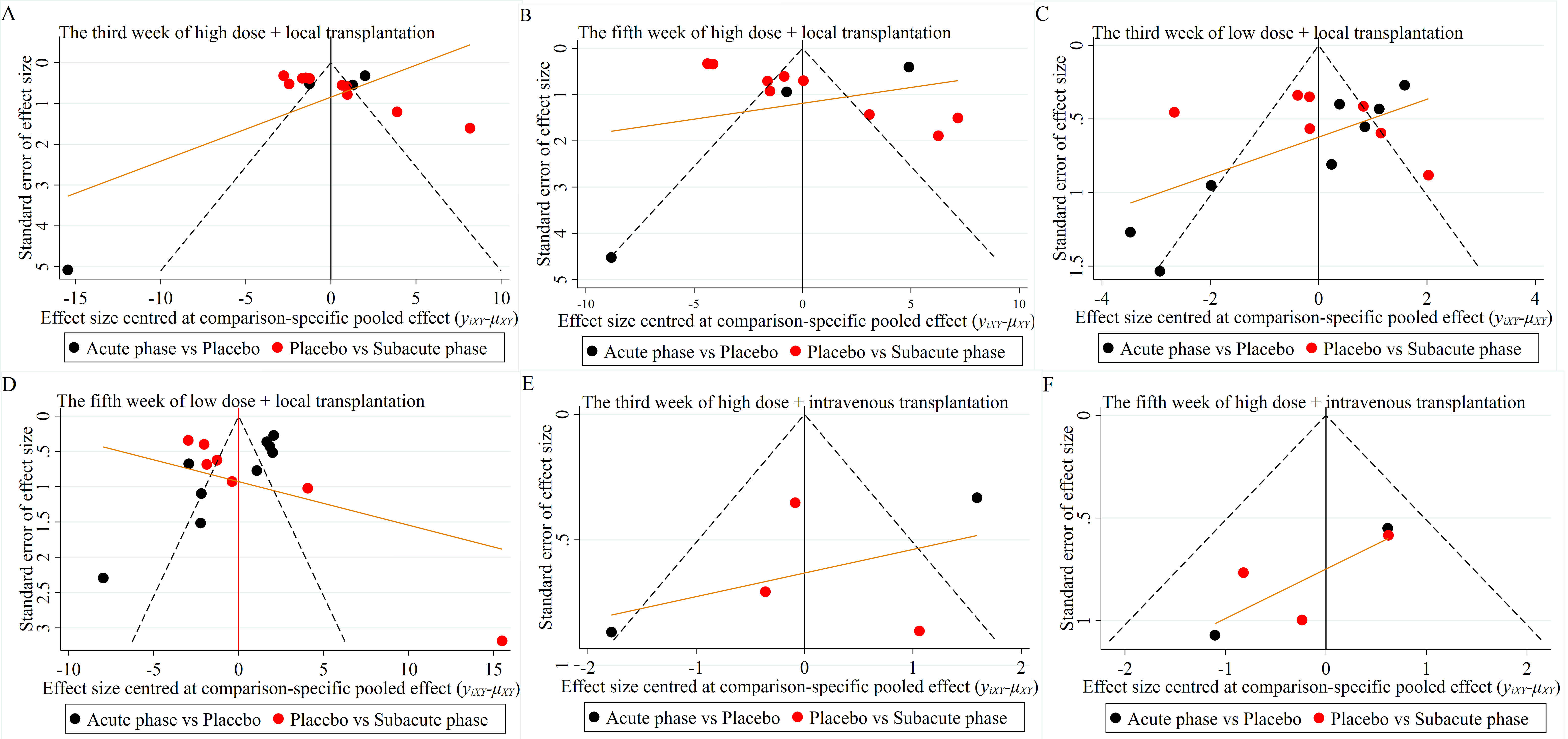
**

**Supplementary Material Figure 6: Comparison-corrected funnel plots of optimal transplantation dose.**
